# Identifying suitable methods for evaluating the sterilizing effects of pyriproxyfen on adult malaria vectors: a comparison of the oviposition and ovary dissection methods

**DOI:** 10.1186/s12936-024-04983-2

**Published:** 2024-05-24

**Authors:** Alesha Myers, Josias Fagbohoun, Georgine Houetohossou, Boris Ndombidje, Renaud Govoetchan, Damien Todjinou, Corine Ngufor

**Affiliations:** 1 London School of Hygiene and Tropical Medicine (LSHTM), London, WC1E 7HT UK; 2 Centre de Recherches Entomologiques de Cotonou (CREC), Cotonou, Benin; 3 Panafrican Malaria Vector Research Consortium (PAMVERC), Cotonou, Benin; 4 African Institute for Research in Infectious Diseases (AIRID), Cotonou, Benin

**Keywords:** Pyriproxyfen, Bottle bioassays, Pyrethroid-pyriproxyfen nets, Ovary dissection, Oviposition inhibition, Pyrethroid resistance, Pyriproxyfen resistance, Insecticide treated nets, Malaria vectors

## Abstract

**Background:**

Nets containing pyriproxyfen, an insect growth regulator that sterilizes adult mosquitoes, have become available for malaria control. Suitable methods for investigating vector susceptibility to pyriproxyfen and evaluating its efficacy on nets need to be identified. The sterilizing effects of pyriproxyfen on adult malaria vectors can be assessed by measuring oviposition or by dissecting mosquito ovaries to determine damage by pyriproxyfen (ovary dissection).

**Method:**

Laboratory bioassays were performed to compare the oviposition and ovary dissection methods for monitoring susceptibility to pyriproxyfen in wild malaria vectors using WHO bottle bioassays and for evaluating its efficacy on nets in cone bioassays. Blood-fed mosquitoes of susceptible and pyrethroid-resistant strains of *Anopheles gambiae *sensu lato were exposed to pyriproxyfen-treated bottles (100 μg and 200 μg) and to unwashed and washed pieces of a pyriproxyfen long-lasting net in cone bioassays. Survivors were assessed for the sterilizing effects of pyriproxyfen using both methods. The methods were compared in terms of their reliability, sensitivity, specificity, resources (cost and time) required and perceived difficulties by trained laboratory technicians.

**Results:**

The total number of *An. gambiae s.l.* mosquitoes assessed for the sterilizing effects of pyriproxyfen were 1745 for the oviposition method and 1698 for the ovary dissection method. Fertility rates of control unexposed mosquitoes were significantly higher with ovary dissection compared to oviposition in both bottle bioassays (99–100% vs. 34–59%, P < 0.05) and cone bioassays (99–100% vs. 18–33%, P < 0.001). Oviposition rates of control unexposed mosquitoes were lower with wild pyrethroid-resistant *An. gambiae s.l*. Cové, compared to the laboratory-maintained reference susceptible *An gambiae *sensu stricto Kisumu (18–34% vs. 58–76%, P < 0.05). Sterilization rates of the Kisumu strain in bottle bioassays with the pyriproxyfen diagnostic dose (100 μg) were suboptimal with the oviposition method (90%) but showed full susceptibility with ovary dissection (99%). Wild pyrethroid-resistant Cové mosquitoes were fully susceptible to pyriproxyfen in bottle bioassays using ovary dissection (> 99%), but not with the oviposition method (69%). Both methods showed similar levels of sensitivity (89–98% vs. 89–100%). Specificity was substantially higher with ovary dissection compared to the oviposition method in both bottle bioassays (99–100% vs. 34–48%) and cone tests (100% vs.18–76%). Ovary dissection was also more sensitive for detecting the residual activity of pyriproxyfen in a washed net compared to oviposition. The oviposition method though cheaper, was less reliable and more time-consuming. Laboratory technicians preferred ovary dissection mostly due to its reliability.

**Conclusion:**

The ovary dissection method was more accurate, more reliable and more efficient compared to the oviposition method for evaluating the sterilizing effects of pyriproxyfen on adult malaria vectors in susceptibility bioassays and for evaluating the efficacy of pyriproxyfen-treated nets.

**Supplementary Information:**

The online version contains supplementary material available at 10.1186/s12936-024-04983-2.

## Background

Insecticide-treated nets (ITNs) are a major pillar in the prevention and control of malaria with over 2.5 billion nets distributed worldwide since 2000, contributing substantially to reductions in malaria burden [1]. The impact of ITNs for malaria control is threatened by the spread and increasing intensity of resistance to pyrethroids which were for almost three decades the only insecticide used on nets [2, 3]. In response to this threat, three new types of insecticide-treated nets (ITNs) containing a mixture of pyrethroids and non-pyrethroid compounds, i.e. the synergist piperonyl butoxide (PBO), the pyrrole chlorfenapyr and the insect growth regulator pyriproxyfen, have been developed for malaria control [4, 5]. Based on evidence from cluster randomized trials (CRT) [6–9], the World Health Organization (WHO) has issued specific recommendations for their use for malaria control over pyrethroid-only ITNs [4]. Following this WHO endorsement, an increased uptake of novel ITNs is already being observed across Africa with several endemic countries shifting away from pyrethroid-only ITNs to dual active ingredient ITNs [10].

As new nets are scaled up, the methods used for evaluating their efficacy and for monitoring susceptibility to the new insecticides in them to help guide decision-making for their deployment must be aligned with their modes of action. The WHO has recently released new guidelines and standard operating procedures for investigating resistance to new public health insecticides using bottle bioassays and for selecting appropriate interventions for different settings [11–14]. Unlike PBO and chlorfenapyr, which provide enhanced mortality of pyrethroid-resistant vector mosquitoes, pyriproxyfen is an insect growth regulator that acts on nets mostly by sterilizing adult female mosquitoes [15, 16]. Based on this mode of action, two different methods have been proposed for monitoring its reproductive effects on adult malaria vector mosquitoes in ITN efficacy studies and susceptibility bioassays: (1) assessment of the effect of pyriproxyfen on mosquito oviposition (oviposition method) [16–19] and (2) dissection of mosquito ovaries to assess their developmental stage (ovary dissection method) [20–24].

The oviposition method measures the direct impact of pyriproxyfen on mosquito offspring by holding exposed blood-fed mosquitoes in oviposition chambers to allow them to lay eggs and determining the proportional reduction in numbers laying, number of eggs per female and number of larvae per female relative to unexposed mosquitoes. Earlier studies evaluating the impact of pyriproxyfen on ITNs used this method to demonstrate and measure its sterilizing properties on malaria vectors [16–18]. The recent WHO standard operating procedure for bottle bioassays to investigate malaria vector susceptibility to the sterilizing properties of pyriproxyfen requires that blood-fed mosquitoes are exposed to pyriproxyfen in bottles treated at a diagnostic concentration of 100 μg per bottle and its sterilizing effects assessed by the oviposition method to determine the reduction in the proportion of mosquitoes laying eggs over a total of 7 days post-exposure [14]. The major challenge behind the oviposition method is the poor oviposition rate usually observed with mosquito strains that are not well adapted to rearing under laboratory conditions [18, 19, 24], making the interpretation of results sometimes impossible. The ovary dissection method on the other hand is based on the physiological impact of pyriproxyfen on the ovarian development of adult female mosquitoes leaving them visibly damaged and halting their follicular maturation process [20]. Using this method, mosquito ovaries are typically dissected under a microscope three days after exposure to pyriproxyfen, and their developmental stage is assessed to determine their fertility status. Mosquitoes with fully developed ovaries containing viable eggs are considered fertile [25] and the proportional reduction in numbers of fertile mosquitoes relative to the control is assessed as a measure of the sterilizing effect. The number of viable eggs found in the dissected ovaries of each mosquito is sometimes counted to determine the overall reduction in the fecundity of mosquitoes [24].

The present study was designed to compare the suitability and practicality of the oviposition and ovary dissection methods for monitoring the susceptibility of wild malaria vectors to pyriproxyfen in WHO bottle bioassays and for evaluating the bioefficacy of pyriproxyfen on mosquito nets using WHO cone bioassays. The methods were compared in terms of their capacity to detect mosquitoes that had been exposed to pyriproxyfen in both bioassay types. Blood-fed pyrethroid susceptible and pyrethroid-resistant strains of *Anopheles gambiae *sensu lato (*s.l*.) were exposed to pyriproxyfen in WHO bottle bioassays and to unwashed and washed pyriproxyfen-treated long-lasting nets in WHO cone bioassays, and the sterilizing effects of pyriproxyfen assessed using both methods. A questionnaire was administered to a group of well-trained technicians at the CREC-LSHTM Facility in Benin to assess their preference and perception of the complexity of the ovary dissection method relative to the oviposition method.

## Methods

### Mosquito strains

The laboratory bioassays were performed with a laboratory-reared susceptible strain and two laboratory-reared pyrethroid-resistant strains of *An. gambiae*. All three strains are maintained at CREC/LSHTM insectary in Cotonou, Benin. The characteristic of each strain is described below.

- Kisumu strain: *An. gambiae *sensu stricto (*s.s*.), an insecticide-susceptible reference strain originating from the Kisumu area in Kenya and was colonized at the CREC/LSHTM insectary.
- Akron strain: *Anopheles coluzzii*, a pyrethroid and carbamate-resistant strain originating from Akron (9° 19′ N2° 18′ E), Southern Benin, and maintained at CREC/LSHTM insectary. Resistance is mediated by target site mutations (L1014F *kdr* and *Ace-1R*) and overexpressed cytochrome P450 enzymes [26].
- Covè strain: *An. gambiae s.l*. is an insecticide-resistant field strain which are F1 progeny of mosquitoes collected from the CREC/LSHTM field station in Covè (7° 14′ N2° 18′ E), southern Benin. The strain exhibits a high frequency of resistance to pyrethroids and organochlorines but remains susceptible to other insecticide classes [27]. The strain is composed of a mixture of *An. coluzzii* and *An. gambiae s.s*.. Resistance is mediated by a target site *kdr* mutation (L1014F) and overexpressed cytochrome P450 enzymes [28].

### WHO insecticide susceptibility tube bioassays

WHO tube tests [29] were performed during the study to confirm the susceptibility status of each mosquito strain to pyrethroids, carbamates and organophosphates. Filter papers treated with 0.05% deltamethrin, 4% DDT, 5% malathion and 0.1% bendiocarb, obtained from Universiti Sains Malaysia were used for testing. PBO pre-exposure bioassays were also performed to investigate the involvement of overexpressed cytochrome P450 enzymes in pyrethroid resistance. Unfed 2–5 days old mosquitoes of each strain were exposed for 1h to the insecticide-treated papers and mortality was recorded 24h later. Approximately 100 mosquitoes were tested for each insecticide treatment in four replicates of 25 mosquitoes. Control mosquitoes were exposed to untreated papers. The bioassays were performed at a temperature of 27 °C ± 2 °C and a relative humidity of 75% ± 10%.

### Mosquito feeding

Preliminary studies using 3-min cone bioassays with pyriproxyfen-treated nets showed no difference in sterilization outcomes for *An. gambiae* mosquitoes that were blood-fed before exposure compared to those that were blood-fed after exposure (Fig. S1). However, when mosquito feeding was performed post-exposure, blood-feeding rates were lower, and more mosquitoes escaped or died before the assessment of sterilizing effects. Hence, all mosquitoes used for bioassays comparing the oviposition and dissection methods in this study were blood-fed before exposure. These mosquitoes were inseminated 5–8 days old females that were blood-fed for 1 h with a live rabbit. Blood-feeding was performed 2 h before exposure for mosquitoes tested in bottle bioassays in line with WHO protocols [30] and 12–18 h before exposure for mosquitoes tested in WHO cone tests. After blood-feeding, mosquitoes were maintained in cages at 27 ˚C ± 2 ˚C and 75% ± 10% relative humidity and provided 10% glucose solution until testing.

### Testing of mosquitoes in bottle bioassays and cone tests

**Fig. 1:**
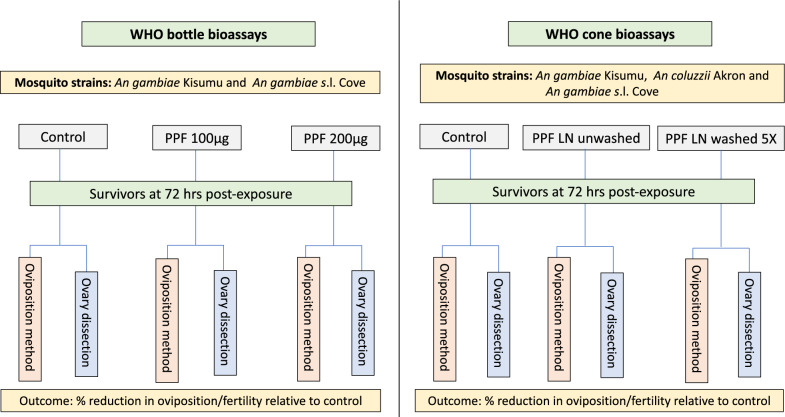
Testing scheme for evaluating the sterilizing effects of pyriproxyfen in WHO bottle bioassays and cone tests. Mosquitoes were exposed to pyriproxyfen in bottle bioassays at doses of 100 μg and 200 μg per bottle and in cone bioassays to unwashed and 5 times washed pyriproxyfen long-lasting nets. Survivors from the individual treatments in each bioassay were divided into two groups; one group was assessed for sterilization by the oviposition method and the other by the ovary dissection method. PPF LN: pyriproxyfen long-lasting net

WHO bottle bioassays were performed with the susceptible *An. gambiae* Kisumu strain and the pyrethroid-resistant *An. gambiae* Cové strain while the WHO cone tests were performed with all three test strains, i.e., *An. gambiae* Kisumu. pyrethroid-resistant *An. gambiae* Cové, and pyrethroid-resistant *An. coluzzii* Akron. Figure 1 summarizes the testing scheme of the study, demonstrating how the blood-fed mosquitoes of each strain were subjected to each type of bioassay and survivors were assessed for the sterilizing effects of pyriproxyfen using the oviposition and ovary dissection methods.

#### WHO bottle bioassays procedure with pyriproxyfen

To compare the oviposition and ovary dissection methods for monitoring the susceptibility of malaria vector mosquitoes to pyriproxyfen using bottle bioassays, blood-fed mosquitoes were exposed to bottles treated with pyriproxyfen at the WHO diagnostic dose of 100 µg/bottle and at a higher dose of 200 µg/bottle (Fig. 1) following WHO protocols [30]. The latter was included to determine whether a higher dose might be more suitable for monitoring susceptibility to pyriproxyfen. Stock solutions were prepared by diluting the technical grade of pyriproxyfen (Disease Control Technologies LTD) in acetone. A total of 24 replicate bottles were prepared per pyriproxyfen dose throughout the study. Each bottle was manually coated using a tube roller with 1 ml of the stock solution in line with WHO procedures [14, 30]. Control bottles treated with acetone alone were also prepared. After coating, the bottles were allowed to dry for 2 h before bottle bioassays were performed. A total of ~ 600 blood-fed mosquitoes of the susceptible *An. gambiae* Kisumu and ~ 300 blood-fed wild F1 pyrethroid-resistant *An. gambiae* Cové were exposed for 1 h to each pyriproxyfen dose in cohorts of 25 per bottle. After exposure, they were held in net-covered plastic cups and provided with 10% glucose solution. Mosquito mortality was recorded at 72 h post-exposure. All bottle bioassays were performed at 27 ˚C ± 2 ˚C and 75% ± 10% relative humidity. Mosquitoes that survived at 72 h post-exposure were divided into two groups for assessment of the reproductive effects of pyriproxyfen using the oviposition and ovary dissection methods (Fig. 1).

#### WHO cone bioassays procedure with pyriproxyfen long-lasting nets

To compare the oviposition and ovary dissection methods for evaluating the impact of pyriproxyfen-treated nets on adult malaria vectors using WHO cone bioassays, blood-fed mosquitoes of all three strains (Kisumu, Cové and Akron) were exposed to unwashed and washed (5 times) net pieces of a pyriproxyfen-only treated net developed by Disease Control Technologies, USA*.* The net consisted of polyethylene fibres incorporated with pyriproxyfen at a concentration of 5 g/Kg. Washing followed WHO protocol [31]; net samples measuring 25cm × 25 cm were placed in a 1 L bottle containing a standardized soap solution and shaken for 10 min in a shaker bath set at 30 °C at 155 movements per minute. The samples were then rinsed twice for 10 min in clean water before being dried at room temperature. A washing interval of 3 days was applied between washes based on previous studies that detected a regeneration time of 3 days for pyriproxyfen in these nets [18]. Four unwashed and four 5 times washed pieces (25 cm × 25 cm) of the pyriproxyfen long-lasting net were prepared for cone bioassays. The net pieces were stored in an incubator at 30 °C and 75–85% relative humidity between washes and between bioassays. Approximately 200–320 blood-fed mosquitoes of each strain were exposed for 3 min in WHO cone bioassays to each net treatment and the control untreated nets in replicates of 5 mosquitoes per cone and 10–15 cones per net piece. Mosquito mortality was recorded at 72 h post-exposure. All bioassays were performed at 27 °C  ± 2 °C and 75% ± 10% relative humidity. Mosquitoes that survived at 72 h post-exposure were also divided into two groups for assessment of the reproductive effects of pyriproxyfen using oviposition and ovary dissection methods.

### Assessment of reproductive effects by oviposition

Mosquito egg-laying chambers were prepared for each exposed mosquito that was set aside for assessment of the sterilizing effects of pyriproxyfen by the oviposition method. The oviposition chamber consisted of 50 ml of deionized water held in a 200 ml net-covered plastic cup (Fig. S2). After recording mortality at 72 h following exposure in bottle bioassays or cone tests, each mosquito was held in its oviposition chamber and provided 10% glucose solution using a piece of cotton placed on the net cover. On day 4 post chambering, mosquitoes were inspected in their oviposition chambers for the presence or absence of eggs and the numbers ovipositing was recorded. Oviposition chambers were held at 27 °C ± 2 °C and 75% ± 10% relative humidity.

Based on preliminary studies that demonstrated a substantial initial impact of pyriproxyfen on the proportion of ovipositing adult female mosquitoes, the reduction in the proportion of ovipositing pyriproxyfen-exposed female mosquitoes relative to the control unexposed group (oviposition inhibition) was considered as the final endpoint for the oviposition method. Further assessments of the number of eggs laid and larvae produced were not included in this study. The reproductive impact of each pyriproxyfen dose in bottle bioassays and each pyriproxyfen net treatment in cone bioassays assessed by the oviposition method was therefore measured in terms of the following:

- Oviposition rate for each treatment and control defined as the proportion of females that laid eggs and calculated as follows:
  $$\mathbf{O}\mathbf{v}\mathbf{i}\mathbf{p}\mathbf{o}\mathbf{s}\mathbf{i}\mathbf{t}\mathbf{i}\mathbf{o}\mathbf{n}\;\mathbf{r}\mathbf{a}\mathbf{t}\mathbf{e}(\mathbf{\%})=\frac{Number\;of\;females\;that\;laid\;eggs}{Total\; number\;of\;chambered\;females} \times 100$$
- Oviposition inhibition for each treatment defined as the proportional reduction in the proportion of females that laid eggs in the treatment relative to the untreated control. Oviposition inhibition was calculated as follows:
  $$\mathbf{O}\mathbf{v}\mathbf{i}\mathbf{p}\mathbf{o}\mathbf{s}\mathbf{i}\mathbf{t}\mathbf{i}\mathbf{o}\mathbf{n}\mathbf{i}\mathbf{n}\;\mathbf{h}\mathbf{i}\mathbf{b}\mathbf{i}\mathbf{t}\mathbf{i}\mathbf{o}\mathbf{n}\left(\mathbf{\%}\right)=1-\frac{Oviposition\;rate \left(\%\right)\;in\;treatment}{Oviposition\;rate \left(\%\right)in\;control} \times 100$$

As per WHO standard procedure for pyriproxyfen susceptibility bottle bioassays, if the oviposition rate was < 30% in the control unexposed group, the test results were discarded and repeated. To better understand the impact of the oviposition rate of control unexposed mosquitoes on the interpretation of results of pyriproxyfen ITN cone bioassays, no cut-off was applied for oviposition rates in control mosquitoes that were tested in the cone bioassays.

### Assessment of reproductive effects by ovary dissection

**Fig. 2:**
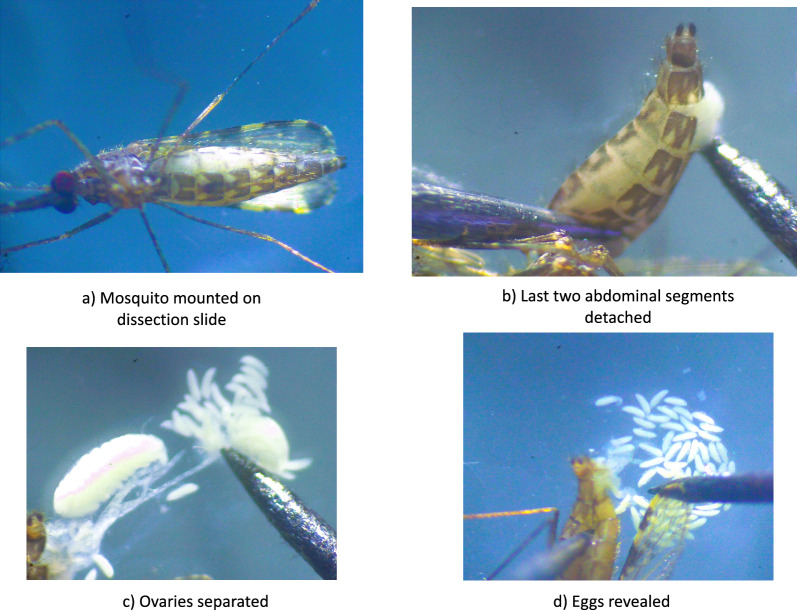
Mosquito ovary dissection procedure. The mosquito was mounted on a dissecting slide (**a**), the last two abdominal segments detached using dissecting needles (**b**), ovaries were separated (**c**) and the eggs revealed (**d**).

**Fig. 3:**
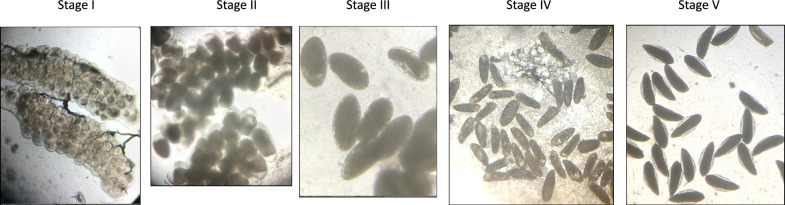
Developmental stages of anopheline ovaries [25] (adapted from [21]). Mosquitoes with fully developed stage V ovaries (elongated boat-shaped eggs with lateral floats) are considered fertile while mosquitoes with stage I–IV ovaries containing underdeveloped eggs are considered infertile.

Ovary dissections were performed under a dissecting microscope at low magnification on day 3 post-exposure. Prior to dissections, mosquitoes were immobilized by holding them at − 20 °C for 5 to 10 min. Each freshly immobilized mosquito was mounted on a dissecting slide with its abdomen pointing to the right. Using dissecting needles and a drop of distilled water, the last two segments of the mosquito’s abdomen were detached to reveal the abdominal contents and the ovaries were dragged into the water and the eggs separated (Fig. 2). The eggs in each mosquito’s ovaries were then observed under a compound microscope (Brunel Microscopes LTD) at 4 × and 10 × magnification to determine their developmental status using Christophers’ stages of egg development classification (Fig. 3). Mosquitoes were classified as ‘fertile’ if eggs had fully developed to stage V (oocyst fills the entire length of the follicle and lateral floats are formed) and ‘infertile’ if eggs had not fully developed and remained in stages I to IV (oocyst occupies 0 to 90% of the follicle, follicle is round and lacks floats). Egg development stages and fertility status were recorded for each mosquito, and several images were taken of the eggs. As with the oviposition method, the final endpoint of the ovary dissection method was the reduction in the proportion of fertile females with mature stage V ovaries relative to the control unexposed group. Further assessments of the number of viable eggs in each dissected mosquito’s ovaries were not included in this study.

The reproductive impact of each pyriproxyfen bottle or ITN treatment assessed by the ovary dissection method was measured in terms of the following:

- Fertility rate defined as the proportion of fertile mosquitoes in a given treatment or control and calculated as follows:
  $$\mathbf{F}\mathbf{e}\mathbf{r}\mathbf{t}\mathbf{i}\mathbf{l}\mathbf{i}\mathbf{t}\mathbf{y}\;\mathbf{r}\mathbf{a}\mathbf{t}\mathbf{e}(\mathbf{\%})=\frac{Total\;number\;of\;fertile\;females}{Total\;number\;of\;females\;observed} \times 100$$
- Reduction in fertility rate for each treatment defined as the proportional reduction in the fertility rate of a given treatment relative to the control. Reduction in fertility rate was calculated as follows:
  $$\mathbf{R}\mathbf{e}\mathbf{d}\mathbf{u}\mathbf{c}\mathbf{t}\mathbf{i}\mathbf{o}\mathbf{n}\;\mathbf{i}\mathbf{n}\mathbf{f}\mathbf{e}\mathbf{r}\mathbf{t}\mathbf{i}\mathbf{l}\mathbf{i}\mathbf{t}\mathbf{y}\left(\mathbf{\%}\right)=1-\frac{Fertility\; rate \left(\%\right) in\; treatment}{Fertility\;rate \left(\%\right) in\; control} \times 100$$

### Timeline for assessment of sterilizing effects

**Fig. 4:**
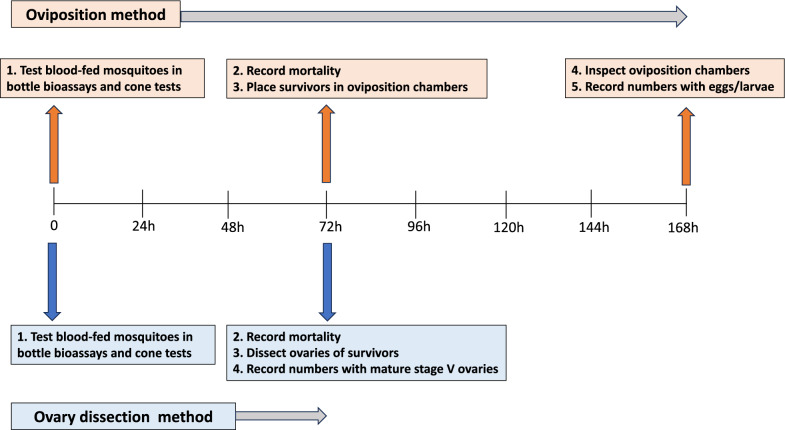
Timeline for assessment of reproductive effects post-exposure in bioassays by oviposition and ovary dissection methods

Figure 4 presents the timeline for the assessment of the sterilizing effects by the oviposition and ovary dissection methods. Mosquito mortality was recorded for 72 h post-exposure in both bottle bioassays and cone tests. Survivors from each treatment were divided into two groups, one group subjected to assessment of sterilization by the oviposition method and the other group by the ovary dissection method as described in Fig. 1. The minimum time required to generate data on reproductive outcomes post-exposure in bioassays was 168 h for the oviposition method and 72 h for the ovary dissection method.

### Comparison of practicality and resources required

Further studies were performed to compare the oviposition and ovary dissection methods in terms of the time taken to complete the assay and obtain final endpoints, resources, number of staff required and the perceived complexity and challenges of the method by fully trained staff. To compare the resources required in terms of costs, the equipment, materials and staff time for each method were listed and their actual costs were obtained from financial records of the CREC-LSHTM Facility in Benin. To compare the time taken, the average time in days between obtaining live mosquitoes from the bioassays and obtaining the complete results of the reproductive outcome was measured for each bioassay round. To assess the perceived difficulty of each method and the interpretation of its results, a short questionnaire was administered to 10 trained laboratory staff at the CREC-LSHTM Facility with routine responsibility for conducting these bioassays. Staff were asked to rate each method on a scale of 1–5 in terms of the (1) efforts required, (2) complexity, (3) difficulty and (4) resources required in terms of space and time. Staff were also asked to state what method they preferred and to provide reasons why.

### Data analysis

Susceptibility tube test data for the test strains was analysed by calculating the pooled means and comparing this against WHO-defined cut-offs for susceptibility. Data on the proportion of females laying eggs (oviposition method) and the proportion of fertile females (dissection method) was compared between treatments using logistic regression with replicate rounds of bioassays included as fixed effects (STATA version 17). To compare the two methods quantitatively and assess their accuracy, the sensitivity, specificity, positive predictive value (PPV) and negative predictive value (NPV) were estimated for each method and each strain. The calculations were performed using formulas in Table 1.

**Table 1:** Calculation of sensitivity and specificity of oviposition and ovary dissection methods

	PPF-exposed	Non-exposed	
Infertile	(a) number of non-laying/infertile females exposed to PPF *(true positive)*	(b) number of non-laying/infertile control females *(false positive)*	${\varvec{P}}{\varvec{P}}{\varvec{V}}={\varvec{a}}/({\varvec{a}}+{\varvec{b}})$
Fertile	(c) number of ovipositing/fertile females exposed to PPF *(false positive)*	(d) number of ovipositing/fertile control females *(true negative)*	${\varvec{N}}{\varvec{P}}{\varvec{V}}={\varvec{d}}/({\varvec{c}}+{\varvec{d}})$
	${\varvec{S}}{\varvec{e}}{\varvec{n}}{\varvec{s}}{\varvec{i}}{\varvec{t}}{\varvec{i}}{\varvec{v}}{\varvec{i}}{\varvec{t}}{\varvec{y}}={\varvec{a}}/({\varvec{a}}+{\varvec{c}})$	${\varvec{S}}{\varvec{p}}{\varvec{e}}{\varvec{c}}{\varvec{i}}{\varvec{f}}{\varvec{i}}{\varvec{c}}{\varvec{i}}{\varvec{t}}{\varvec{y}}={\varvec{b}}/({\varvec{b}}+{\varvec{d}})$	

PPV: Positive Predictive Value, NPV: Negative Predictive Value, PPF: pyriproxyfen

## Results

### Susceptibility of mosquito strains

**Fig. 5:**
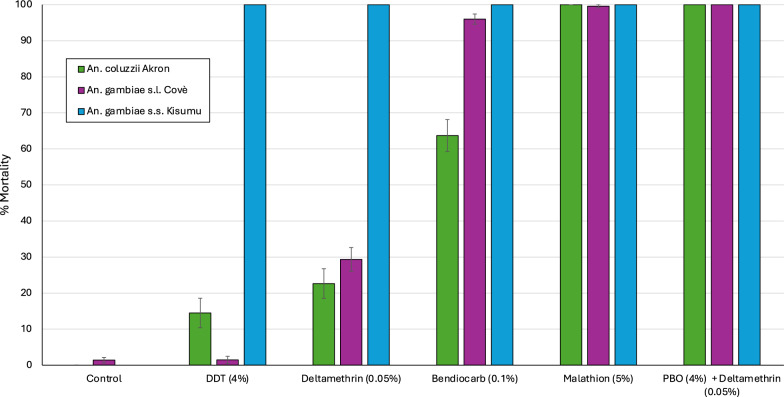
Mortality of *An. gambiae* Kisumu, *An. gambiae* Cové and *An. coluzzii* Akron strains in WHO susceptibility tube tests. Approximately 100 mosquitoes were exposed to each insecticide treatment in replicates of 25 per tube

Mosquito mortality in WHO susceptibility tube tests with the *An. gambiae* Kisumu strain was 100% with all insecticides tested confirming the susceptibility of this strain (Fig. 5). With *An. gambiae* Cové, mosquito mortality was < 30% with DDT and deltamethrin thus confirming resistance to organochlorines and pyrethroids in this vector population. The *An. coluzzii* Akron strain showed resistance to DDT, deltamethrin and bendiocarb as mortality rates were < 70% with all three insecticides. Susceptibility to pyrethroids increased to 100% with the Cové and Akron pyrethroid-resistant strains when pre-exposed to PBO thus demonstrating the full involvement of overexpressed P450s in pyrethroid resistance in both strains.

### WHO pyriproxyfen bottle bioassay results

A total of 1519 insecticide-susceptible *An. gambiae* Kisumu and 909 wild pyrethroid-resistant *An. gambiae* Cové females were tested in the bottle bioassays.

### Bottle bioassay mortality results

Mortality at 72 h post-exposure was higher with the susceptible Kisumu strain (41–42%) compared to the pyrethroid-resistant Cové strain (~ 20%) (Table 2) at both doses of pyriproxyfen. For both strains, there was no evidence of an increase in mortality as the dose of pyriproxyfen increased from 100 μg or 200 μg.

**Table 2:** Mortality rates (72h) of susceptible *An. gambiae* Kisumu strain and pyrethroid-resistant *An. gambiae* Cové strain exposed to pyriproxyfen in bottle bioassays

Strain	Treatment	N blood-fed females exposed	N dead at 72 h	N alive at 72 h	% Mortality	(95% CI)
Kisumu	Control	298	23	275	7.72	4.69–10.75
	PPF 100 μg	612	250	362	40.85	36.96–44.74
	PPF 200 μg	609	257	352	42.2	38.28–46.12
Cové	Control	200	6	194	3	0.64–5.36
	PPF 100 μg	328	64	264	19.51	15.22–23.80
	PPF 200 μg	380	74	306	19.47	15.49–23.46

PPF: Pyriproxyfen

### Bottle bioassay reproductive outcomes

Following 72 h mortality readings in bottle bioassays, the number of surviving mosquitoes assessed for reproductive effects was 981 for the susceptible Kisumu strain and 764 for the pyrethroid-resistant Cové strain. Survivors were divided into two almost equal groups and one group assessed for the reproductive effects using the oviposition method and the other group using the ovary dissection method. A summary of the reproductive outcomes with each strain is provided in Table 3 for both oviposition and ovary dissection methods. The fertility rates indicated by the oviposition rate (oviposition method) and proportion of fertile (ovary dissection method) for mosquitoes exposed to the control exceeded 30% for both methods and were substantially higher with the ovary dissection method compared to the oviposition method for both the susceptible Kisumu strain (99% vs 59%) and the pyrethroid-resistant Cové strain (100% vs. 34%). The sterilizing effect on PPF-exposed mosquitoes was generally higher with the dissection method compared to the oviposition method (99% vs. 84–90% for Kisumu and 96–99% vs. 66–69% for Cové). This difference was greater with the wild pyrethroid-resistant Cové strain compared to the susceptible Kisumu strain. With the oviposition method, the sterilizing effect was less than 90% with both strains but exceeded 98% with both strains when the ovary dissection method was used. For both strains and both methods, the difference in sterilizing effects appeared to be similar between the higher and lower doses of pyriproxyfen, though a slight decrease was observed at the higher pyriproxyfen dose of 200 μg with the oviposition method. For both methods, there was no evidence of an increase in sterilizing effects as the dose of pyriproxyfen increased from 100 μg per bottle to 200 μg per bottle.

**Table 3:** WHO bottle bioassay results on sterilizing effects of pyriproxyfen on susceptible *An. gambiae* Kisumu and pyrethroid-resistant *An. gambiae* Cové mosquitoes assessed by oviposition vs. ovary dissection methods

	Kisumu			Cové		
	Control	PPF 100 μg	PPF 200 μg	Control	PPF 100 μg	PPF 200 μg
*Oviposition method*						
Total number of females observed	130	182	175	100	132	154
Number of females laying eggs	75	10	15	34	14	18
Oviposition rate (%)	57.69	5.49	8.57	34	10.61	11.69
95% Confidence Intervals	49.20–66.19	2.18–8.81	4.42–12.72	24.72–43.28	5.35–15.86	6.61–16.76
Oviposition inhibition (%)	–	89.63	83.83	–	68.81	65.62
*Ovary Dissection*						
Total number of blood-fed females dissected	137	180	177	94	132	152
Total number of infertile females (stage I–IV)	1	178	176	0	131	146
Total number of fertile females (stage V)	136	2	1	94	1	6
Proportion of fertile females (%)	99.27	1.11	0.56	100	0.76	3.95
95% Confidence Intervals	97.84–100	0–2.64	0–1.67	98–100	0–2.24	0.85–7.04
Reduction in fertility per female dissected (%)	–	98.88	99.43	–	99.23	96.01

PPF: Pyriproxyfen

### WHO pyriproxyfen ITN cone bioassay results

A total of 902 blood-fed susceptible *An. gambiae* Kisumu, 607 blood-fed pyrethroid-resistant *An. gambiae* Cové and 726 blood-fed *An. coluzzii* Akron mosquitoes were exposed to the pyriproxyfen long-lasting nets in three-minute cone bioassays (Table 4).

**Table 4:** Mortality of susceptible *An. gambiae* Kisumu, pyrethroid resistant *An gambiae* Cové, and pyrethroid resistant *An. coluzzii* Akron females exposed PPF long-lasting net in WHO cone bioassays

Strain	Treatment	N blood-fed females exposed	N dead	N alive at 72h	% Mortality	(95% CI)
Kisumu	Control	265	19	246	7.17	4.06–10.28
	PPF LN 0W	317	89	228	28.08	23.13–33.02
	PPF LN 5W	320	90	230	28.13	23.20–33.05
Cové	Control	175	17	158	9.71	5.33–14.10
	PPF LN 0W	203	82	121	40.39	33.64–47.14
	PPF LN 5W	229	57	172	24.89	19.29–30.49
Akron	Control	185	7	178	3.78	1.03–6.53
	PPF LN 0W	270	52	218	19.26	14.56–23.96
	PPF LN 5W	271	43	228	15.87	11.52–20.22

PPF LN:  Pyriproxyfen long-lasting net, 0W: unwashed, 5W: washed 5 times

#### Cone bioassay mortality results

Mortality with the control was < 10% across all three strains. Mortality with the PPF-treated nets was 28% with the Kisumu strain, 25–40% with the Cové strain and 16–19% with the Akron strain. There was evidence of a decrease in mosquito mortality with the washed nets compared to the unwashed nets for the insecticide-resistant Cové (40% to 25%) and Akron (19% to 16%) strains.

#### Cone bioassay reproductive outcomes

A total of 666 *An. gambiae* Kisumu, 449 *An. gambiae* Cové and 583 *An. coluzzii* Akron mosquitoes that remained alive after the cone bioassays with the pyriproxyfen long-lasting net were observed for the sterilizing effects using the oviposition and ovary dissection methods. Table 5 summarizes the results obtained with each strain using each method. As observed in the bottle bioassays, the fertility rates of mosquitoes exposed to the control untreated net were generally lower with the oviposition method (18–76% oviposition rate) compared to the dissection method (100% fertile) across all three strains tested (P < 0.05). Control oviposition rates were also higher with the susceptible Kisumu strain (76%) compared to the pyrethroid-resistant Akron and Cové strains (18–33%, P < 0.05). Mosquito sterilization was very high with the unwashed pyriproxyfen nets across all three strains and did not differ substantially between the oviposition (90–98% oviposition inhibition) and ovary dissection method (89–100% reduction in fertility). Sterilization reduced significantly with the washed pyriproxyfen net across all strains tested but was consistently higher with the ovary dissection method compared to the oviposition method, especially with the pyrethroid-resistant strains (65% vs. 45% with Kisumu, 51% vs. 7.4% with Cové strain and 33% vs. 0% with Akron strain, P < 0.05).

**Table 5:** WHO cone bioassay results on sterilizing effects of pyriproxyfen long-lasting nets on susceptible *An. gambiae* Kisumu, pyrethroid-resistant *An. gambiae* Cové and pyrethroid-resistant *An. coluzzii* mosquitoes assessed by oviposition and ovary dissection methods

	Kisumu			Cové			Akron		
	Control	PPF LN 0W	PPF LN 5W	Control	PPF LN 0W	PPF LN 5W	Control	PPF LN 0W	PPF LN 5W
*Oviposition method*									
Total number of females observed	130	120	100	80	58	90	94	100	110
Number of females laying eggs	99	2	42	14	1	15	31	2	41
Oviposition rate (%)	76.15	1.67	42	17.5	1.72	16.67	32.98	2	37.27
95% Confidence Intervals	68.83–83.48	0–3.96	32.33–51.67	9.17–25.83	0–5.07	8.97–24.37	23.47–42.48	0–4.74	28.24–46.31
Oviposition inhibition (%)	–	97.81	44.74	–	90.42	7.41	–	93.94	− 12.95
*Dissection method*									
Total number of blood-fed females dissected	110	108	98	78	61	82	80	99	100
Total number of infertile females (stage I–IV)	0	108	64	0	60	42	0	88	33
Total number of fertile females (stage V)	110	0	34	78	1	40	80	11	67
Proportion of fertile females (%)	100	0	34.69	100	1.64	48.78	100	11.11	67
95% Confidence Intervals	98–100	0–5	25.27–44.12	98–100	0–4.83	37.96–59.60	98–100	4.92–17.30	57.78–76.22
Reduction in fertility per female dissected (%)	–	100	64.96	–	98.36	51.22	–	88.89	33

PPF LN: Pyriproxyfen long-lasting net, 0W: unwashed, 5W: washed 5 times

### Sensitivity and specificity of methods

As the reduction in oviposition or fertility rate per female observed and per female dissected is largely different, sensitivity and specificity were calculated to compare both methods in the bottle bioassays (Table 6) and cone tests (Table 7). For cone tests, only mosquitoes exposed to the unwashed pyriproxyfen net were considered for this comparison given that the dose of pyriproxyfen in the washed net was unknown. In bottle bioassays, both oviposition and ovary dissection methods showed high sensitivity being capable of correctly identifying 93% of PPF-exposed *An. gambiae* Kisumu mosquitoes as infertile and 89% of PPF exposed *An. gambiae* Cové mosquitoes as sterile (Table 6). Similar trends in sensitivity results were obtained in cone bioassays with 98% of PPF-exposed mosquitoes of each strain correctly identified as infertile by the oviposition method and 89–100% of PPF-exposed mosquitoes correctly identified as infertile by the dissection method (Table 7).

**Table 6:** Sensitivity and specificity of the oviposition and ovary dissection methods in pyriproxyfen susceptibility bottle bioassays

	Kisumu			Cové		
	PPF-exposed	Unexposed		PPF-exposed	Unexposed	
*Oviposition method*						
Infertile (no eggs)	332	55	PPV: 86%	254	66	PPV: 79%
Fertile (laid eggs)	25	75	NPV: 75%	32	34	NPV: 52%
	Sensitivity: 93%	Specificity: 58%		Sensitivity: 89%	Specificity:34%	
*Dissection method*						
Infertile (Stage I to IV)	354	1	PPV: 100%	277	0	PPV: 100%
Fertile (Stage V)	3	136	NPV: 98%	7	94	NPV: 93%
	Sensitivity: 99%	Specificity: 99%		Sensitivity: 98%	Specificity: 100%	

Susceptible *An. gambiae* Kisumu and pyrethroid-resistant *An. gambiae* Cové mosquitoes were exposed to bottles treated with pyriproxyfen at 100 μg and 200 μg per bottle for 60 min. Results are combined for both doses

PPV: Positive predictive value, NPV: Negative predictive value

**Table 7:** Sensitivity and specificity of the oviposition and ovary dissection methods in cone bioassays with a pyriproxyfen long-lasting net

	Kisumu			Cové			Akron		
	PPF-exposed	Unexposed		PPF-exposed	Unexposed		PPF-exposed	Unexposed	
*Oviposition method*									
Infertile (no eggs)	118	31	PPV: 79%	57	66	PPV: 46%	98	63	PPV: 61%
Fertile (laid eggs)	2	99	NPV: 98%	1	14	NPV: 93%	2	31	NPV: 94%
	Sensitivity: 98%	Specificity: 76%		Sensitivity: 98%	Specificity: 18%		Sensitivity: 98%	Specificity: 33%	
*Dissection method*									
Infertile (Stage I to IV)	108	0	PPV: 100%	60	0	PPV: 100%	88	0	PPV: 100%
Fertile (Stage HV)	0	110	NPV: 100%	1	78	NPV: 99%	11	80	NPV: 88%
	Sensitivity: 100%	Specificity: 100%		Sensitivity: 99%	Specificity: 100%		Sensitivity: 89%	Specificity: 100%	

Susceptible *An. gambiae* Kisumu, pyrethroid-resistant *An. gambiae* Cové and pyrethroid -resistant *An. coluzzii* Akron mosquitoes were exposed for 3 min to pyriproxyfen long-lasting net in cone bioassays. Results were calculated only for mosquitoes that were exposed to the unwashed net

PPV: Positive predictive value, NPV: Negative predictive value

A different outcome was observed with the levels of specificity between the methods. In bottle bioassays, the levels of specificity were substantially lower with the oviposition method (58% with An. gambiae Kisumu and 34% with *An. gambiae* Cové) compared to the ovary dissection method (99% *An. gambiae* Kisumu and 100% with *An. gambiae* Cové). Similar results were obtained in cone tests; specificity was 18–76% with the oviposition method and 100% with the ovary dissection method across all three strains tested (Table 7). This shows that the ovary dissection was more capable of correctly identifying PPF-unexposed mosquitoes as fertile compared to the oviposition method in both types of bioassays. For each bioassay type, the specificity with the oviposition method was substantially lower with the pyrethroid-resistant strains compared to the susceptible *An. gambiae* Kisumu strain (34% vs. 58% in bottle bioassays and 18–33% vs 76% in cone tests). This difference in specificity by mosquito strain was not observed with the ovary dissection method; specificity was 98–100% across all strains in both bottle bioassays and cone tests.

The positive predictive values (PPV) and negative predictive values (NPV) reflect the levels of sensitivity and specificity observed with each method in each bioassay. In bottle bioassays both PPV and NPV values were higher with the ovary dissection method (100% PPV and 93–98% NPV) compared to the oviposition method (79–86% PPV and 52–75%NPV) (Table 6). This shows greater accuracy and precision of the ovary dissection method for measuring the sterilizing effect of pyriproxyfen on adult malaria vectors in the susceptibility bottle bioassays compared to the oviposition method. In cone bioassays, the PPV was substantially higher with the ovary dissection method compared to the oviposition method (100% vs 46–79%) though the NPV did not differ substantially between both methods (88–100% vs. 93–98%) (Table 7). Hence for cone bioassays, the ovary dissection method was also more precise for detecting the sterilizing effect of PPF-treated nets on exposed mosquitoes.

### Practicality, costs and resources

In terms of costs and resources, oviposition is largely inexpensive, with 200 ml plastic cups and netting costing less than £0.5 per chamber. On the other hand, dissection requires a dissecting set, slides and two types of microscopes (compound and dissecting), which on average cost approximately £10,000 in total (Fig. S3). A digital camera costing £750 is also sometimes used with both methods to keep records of mosquito oviposition or ovary status for future consultations which can further increase the cost of each method. The number of staff required was largely indifferent across both methods, and both methods required skilled laboratory technicians. The dissection method however required less staff time given that it took on average 2–3 less days than the oviposition method to obtain the final endpoints of the reproductive outcomes post-exposure. All technicians preferred the ovary dissection method and scored the oviposition method a lower average score of 3 as it was rated more challenging and more demanding in terms of space and time required compared to ovary dissection which was rated an average score of 4. Staff commented on how “chambering requires more time and space to set up one chamber per mosquito and to observe them for egg laying”. Almost all the staff interviewed commented on how “tedious” and “strenuous” the oviposition method was when it included counting of eggs and larvae. This was also stated for the dissection method if they had to count viable eggs found in the dissected ovaries of mosquitoes. One major challenge mentioned with the oviposition method was that bottle bioassays with wild field-collected mosquitoes had to be invalidated a few times and repeated when the oviposition rate in the control was < 30%. As noted by one senior staff “the uncertainty of achieving the minimum required oviposition rate in the control makes planning of tests with the oviposition method very difficult. This does not usually happen with the ovary dissection method”. The staff mentioned finesse, training, and multiple trial runs as critical parameters to perfect the ovary dissection technique.

## Discussion

As the uptake of pyriproxyfen-treated nets for the control of malaria increases, suitable methods for investigating the susceptibility of local vector mosquitoes to the insect growth regulator and for assessing its efficacy on ITNs need to be developed. This study compared two methods for evaluating the sterilizing effects of pyriproxyfen on adult malaria vectors: oviposition and ovary dissection. The results show multiple advantages for using the ovary dissection method over the oviposition method.

To effectively measure the sterilizing effect of pyriproxyfen on exposed adult mosquitoes in bioassays, a substantial level of fertility should be observed in the control unexposed group irrespective of the method used. The new WHO bottle bioassay protocol for monitoring susceptibility to pyriproxyfen recommends a 30% cut-off for oviposition or fertility rate in the unexposed group and tests which do not achieve this need to be considered invalid and repeated [30]. Using the oviposition method, substantially lower oviposition rates were observed with unexposed pyrethroid-resistant mosquitoes compared to the susceptible Kisumu strain in both bottle bioassays ((34% vs. 57%) and cone tests (18–33% vs. 76%). One round of bottle bioassays with the wild Cové mosquitoes had to be repeated due to the oviposition rate of unexposed mosquitoes falling below the 30% cut-off. Oviposition rates of unexposed wild Cové mosquitoes in cone bioassay experiments were also very low (18%). The artificial oviposition chamber therefore did not provide enough stimulus for oviposition for this strain which is not adapted to ovipositing under laboratory conditions compared to the Kisumu strain. This finding corroborates previous studies reporting much lower oviposition rates with pyrethroid-resistant and wild field-collected PPF-unexposed mosquitoes in bioassays and semi-field experimental hut studies with pyriproxyfen-treated nets, sometimes resulting in inconclusive results [18, 19, 24]. Using the ovary dissection method, almost all PPF-unexposed mosquitoes in bottle bioassays (99–100%) and cone tests (100%) were fertile irrespective of the mosquito strain tested. It was thus possible to obtain more interpretable and reliable results with the ovary dissection method compared to the oviposition method. Unlike the oviposition method, fertility rates of PPF-unexposed mosquitoes in the bottle bioassays with the ovary dissection method were very high (99–100%) across all testing rounds; no repetitions were necessary. The ovary dissection method therefore increases the likelihood of obtaining valid results with fewer mosquitoes and bioassay rounds. This is particularly important for pyriproxyfen susceptibility bottle bioassays as these are to be performed with wild field collected anopheline mosquitoes that are usually very challenging to obtain and are less likely to oviposit under laboratory conditions.

The accuracy of a test method is usually defined by its sensitivity and specificity. The high sensitivity observed with both methods in both bottle bioassays (89–93% with oviposition and 99% with ovary dissection) and cone bioassays (98% with oviposition and 89–100% with ovary dissection) shows that they are equally capable of identifying PPF-exposed mosquitoes as infertile. However, the substantially lower levels of specificity observed with the oviposition method compared to the ovary dissection method demonstrates that the oviposition method is less capable of identifying PPF-unexposed mosquitoes as fertile. The findings, therefore, show higher accuracy and precision of the ovary dissection method over the oviposition method for investigating mosquito sterilization by pyriproxyfen and confirm previous studies [24]. In addition, while specificity with the oviposition method was even lower with the pyrethroid-resistant strains compared to the susceptible Kisumu strain, specificity remained high with the ovary dissection method across all mosquito strains tested further demonstrating its potential to more accurately measure the sterilizing effects of pyriproxyfen against wild pyrethroid resistant and field-collected strains.

While pyriproxyfen has been shown to induce reductions in total number of eggs laid and larvae produced by exposed mosquitoes, based on the WHO pyriproxyfen susceptibility bottle bioassay procedure and on preliminary studies that showed a greater initial impact of pyriproxyfen on the proportion of mosquitoes laying eggs, oviposition inhibition was considered as the final endpoint of the oviposition method for this study. This substantially shortened the time required for the assay from ~ 14 days to 7 days. Although it requires less effort to check mosquito oviposition chambers to determine the presence or absence of eggs than to dissect each mosquito under a microscope, this study found that with well-trained technicians, the ovary dissection method took on average 2–3 fewer days to complete compared to the oviposition method. With limited training of technicians and some investment in a dissection microscope, the benefits of the ovary dissection method should, based on its shorter duration, higher accuracy and efficiency, outweigh the oviposition method, especially when large numbers of pyrethroid-resistant and/or wild field mosquitoes need to be assessed for sterilization over multiple rounds of bioassays e.g. pyriproxyfen susceptibility bioassays, evaluation of pyriproxyfen on nets using laboratory bioassays, semi-field experimental hut and ITN insecticidal durability studies. In addition, the ovary dissection method was better for detecting the activity of pyriproxyfen on washed nets in cone bioassays with the pyrethroid-resistant strains indicating its suitability for monitoring the bioefficacy of pyrethroid-pyriproxyfen ITNs as they age under operational use. Based on this finding, the ovary dissection method was chosen for ongoing durability studies of Royal Guard®, a pyrethroid-pyriproxyfen net, distributed in Benin as part of a large-scale randomized controlled trial [21].

The WHO pyriproxyfen bottle bioassay procedure recommends a diagnostic concentration of 100 μg per bottle and a cut-off of 98% oviposition inhibition for determining susceptibility to pyriproxyfen in anopheline mosquitoes [11, 30]. In bottle bioassays with the wild pyrethroid resistant Cové strain using this diagnostic dose, the results showed 69% oviposition inhibition with the oviposition method and 99% reduction in fertility with the ovary dissection method indicating two contrasting outcomes for the same strain; resistance to pyriproxyfen using the oviposition method and susceptibility to pyriproxyfen when the ovary dissection method was used. Using a higher dose of 200 μg per bottle did not change the outcome of the bioassay with the oviposition method suggesting that the 100 μg dose was optimal. However, for pyriproxyfen susceptibility results to be valid, the sterilization rates in the bottle bioassays with the reference susceptible Kisumu strain tested in parallel should ideally be ≥ 98%. This was achieved with the ovary dissection method (99% reduction in fertility) but not with the oviposition method (90% oviposition inhibition). Unlike the ovary dissection method, the oviposition method produced suboptimal outcomes with the reference Kisumu strain which makes the interpretation of the susceptibility results obtained with the wild pyrethroid resistant Cové strain using this method unreliable. The ovary dissection method therefore provided more interpretable susceptibility results in WHO bottle bioassays with the wild pyrethroid-resistant Cové strain compared to the oviposition method.

## Conclusion

This study shows that the ovary dissection method is more efficient, more accurate and more reliable for evaluating the sterilizing effects of pyriproxyfen on adult malaria vectors in bioassays compared to the oviposition method. WHO pyriproxyfen susceptibility bottle bioassays against a wild pyrethroid-resistant *An gambiae s.l.* population were more interpretable with the ovary dissection method compared to the oviposition method. We recommend using ovary dissection over the oviposition method for assessing mosquito sterilization, especially for pyriproxyfen susceptibility bioassays with wild vector mosquitoes and for studies evaluating pyriproxyfen efficacy on ITNs including laboratory bioassays, semi-field experimental hut trials and insecticidal durability studies.

### Supplementary Information

Supplementary materials 1

Supplementary materials 2

Supplementary materials 3

## Data Availability

The datasets used and/or analysed during the current study are available from the corresponding author on reasonable request.

## Abbreviations

PBO: Piperonyl butoxide
PPF: Pyriproxyfen
ITN: Insecticide-treated net
LN: Long-lasting insecticidal nets
WHO: World Health Organization
AI: Active ingredient
PPV: Positive Predictive Value,
NPV: Negative Predictive Value
cRCT: Cluster randomized controlled trial
CREC: Centre de Recherche Entomologique de Cotonou
LSHTM: London School of Hygiene & Tropical Medicine
PAMVERC: Pan African Malaria Vector Research Consortium
AIRID: African Institute for Research in Infectious Diseases
