# Supplementary material for: Identifying suitable methods for evaluating the sterilizing effects of pyriproxyfen on adult malaria vectors: a comparison of the oviposition and ovary dissection methods
Source: Malar J. 2024 May 24;23:164. doi: 10.1186/s12936-024-04983-2 (PMC11127354; doi:10.1186/s12936-024-04983-2)
Supplement: Supplementary file 1 — Supplementary materials 1 [file 12936_2024_4983_MOESM1_ESM.docx]

**Identifying suitable methods for evaluating the sterilising effects of pyriproxyfen on adult malaria vectors: a comparison of the oviposition and ovary dissection methods.**

**Authors:** Alesha Myers^1+^, Josias Fagbohoun^2,3+^, Georgine Houetohossou^2^, Boris Ndombidje^2,3^, Renaud Govoetchan^1,2,3,4^, Damien Todjinou^2,3^, Corine Ngufor^1, 2, 3,4*^

**Supplementary Figure S1:** Oviposition inhibition rates of mosquitoes that were blood-fed before exposure vs. mosquitoes that were blood-fed after exposure. *Anopheles gambiae* Kisumu mosquitoes were exposed for 3 minutes to unwashed and washed (3 times) samples of a pyriproxyfen long-lasting net in cone bioassays and adapted cylinder net tests. Mosquitoes were tested before or after blood-feeding and assessed for the sterilising effects of pyriproxyfen using the oviposition method. The figure presents the Each bar represents a sample size of 50 mosquitoes.
