# Supplementary material for: Identifying suitable methods for evaluating the sterilizing effects of pyriproxyfen on adult malaria vectors: a comparison of the oviposition and ovary dissection methods
Source: Malar J. 2024 May 24;23:164. doi: 10.1186/s12936-024-04983-2 (PMC11127354; doi:10.1186/s12936-024-04983-2)

**Identifying suitable methods for evaluating the sterilising effects of pyriproxyfen on adult malaria vectors: a comparison of the oviposition and ovary dissection methods.**

**Authors:** Alesha Myers^1+^, Josias Fagbohoun^2,3+^, Georgine Houetohossou^2^, Boris Ndombidje^2,3^, Renaud Govoetchan^1,2,3,4^, Damien Todjinou^2,3^, Corine Ngufor^1, 2, 3,4*^

**Supplementary Figure S3:** Materials required for dissection of mosquito ovaries to assess sterilisation by pyriproxyfen


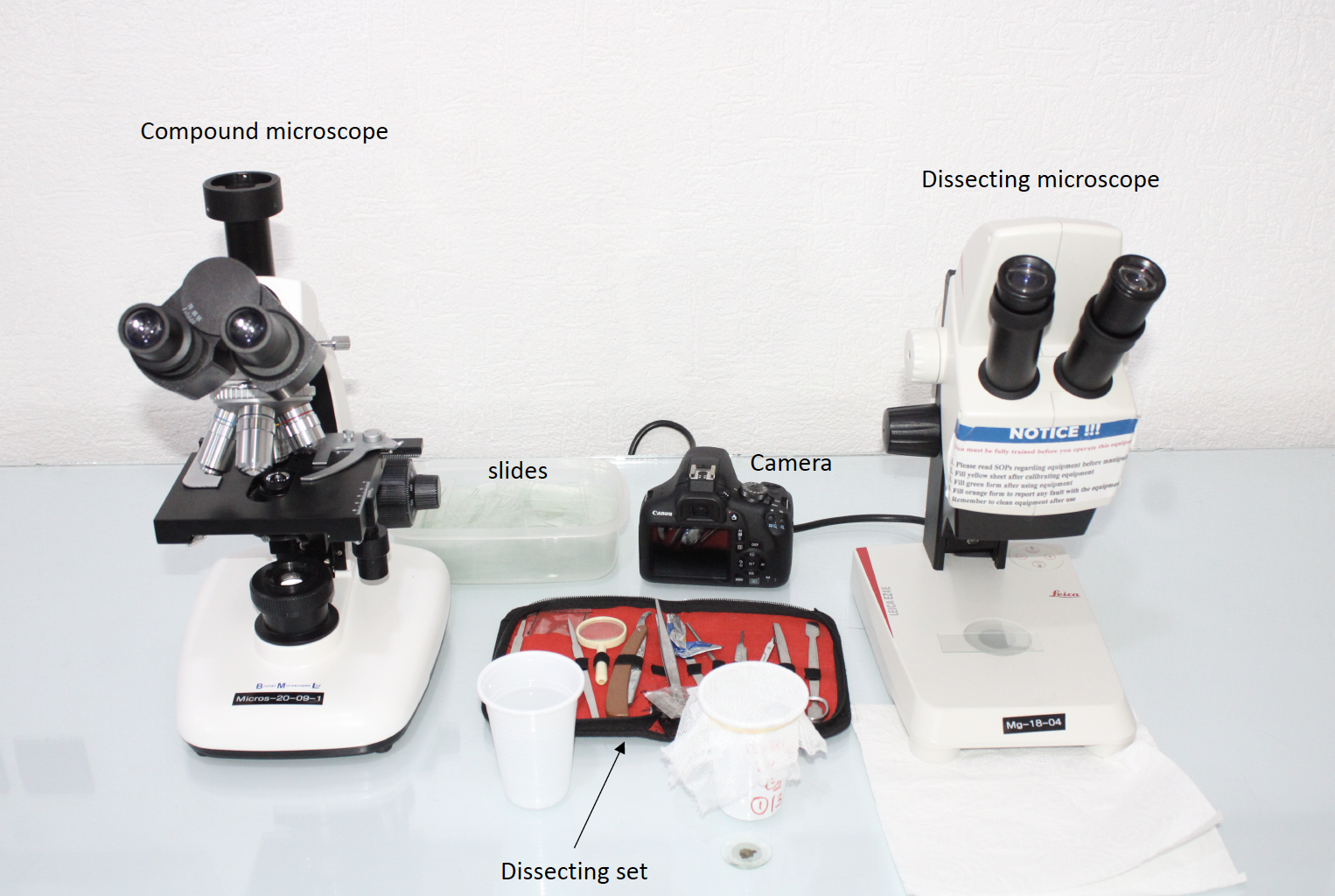

Supplement: Supplementary file 3 — Supplementary materials 3 [file 12936_2024_4983_MOESM3_ESM.docx]
